# Mitophagy and Cellular Homeostasis in Kidney Diseases: Mechanisms and Potential Therapeutics

**DOI:** 10.7150/ijbs.138435

**Published:** 2026-08-12

**Authors:** Hao Lu, Shenyu Yan, Baoyi Shao, Jianbo Guo, Yifan Wang, Rui Hou, Shen Chen, Meicen Wu, Biao Wei, Fei Li, Xiaoming Meng, Haiyong Chen

**Affiliations:** 1 School of Chinese Medicine, Li Ka Shing Faculty of Medicine, The University of Hong Kong, Hong Kong, China.; 2 Department of Nephrology, The University of Hong Kong-Shenzhen Hospital, Shenzhen, China.; 3 Department of Urology, Nanfang Hospital, Southern Medical University, Guangzhou, Guangdong, China.; 4 Inflammation and Immune Mediated Diseases Laboratory of Anhui Province, The Key Laboratory of Anti-Inflammatory of Immune Medicines, Ministry of Education, Anhui Institute of Innovative Drugs, School of Pharmacy, Anhui Medical University, Hefei 230032, China.; 5 Department of Chinese Medicine, The University of Hong Kong-Shenzhen Hospital, Shenzhen, China.

**Keywords:** mitochondrial quality control, mitophagy, kidney disease, targeted therapy

## Abstract

Mitochondrial homeostasis has attracted increasing interest and is now recognized as playing a significant role in both kidney development and the progression of kidney disease. Among these, the latest approach, mitophagy, has been shown to be activated dynamically and reversibly under various physiological conditions, including reactive oxygen stress, nutrient deficiency, and cellular senescence, to maintain mitochondrial homeostasis and function. Moreover, findings indicate that mitophagy can also maintain mitochondrial quality through interactions and mutual regulation with mitochondrial dynamics. Crucially, a growing number of kidney diseases, such as acute kidney injury, diabetic kidney disease, and other chronic kidney diseases, are linked to abnormal levels of mitophagy. In this review, we comprehensively examined the vital role of mitophagy in kidney diseases, discussed the potential of mitophagy-targeted therapies, and described the detailed alterations in specific mitophagy-related proteins associated with kidney diseases.

## 1. Introduction

The global number of people suffering from chronic kidney disease is estimated to be approximately 850 million, and about 4 million people require kidney replacement therapy due to kidney failure. By 2050, chronic kidney disease is projected to rank as the fifth leading cause of death globally 1. The age-standardized prevalence rate of chronic kidney disease among adults worldwide is 14.2% (ranging from 13.4% to 15.2%) 2. Consequently, there is an urgent need to enhance understanding of the mechanisms underlying kidney disease and to develop effective preventive and therapeutic strategies. Recently, studies have provided valuable new insights into various aspects of kidney pathology and treatments 3. Therefore, it is essential to synthesize these findings and to explore innovative approaches for the prevention and treatment of these conditions.

Mitochondria are double-membrane organelles found in most eukaryotic cells. As the main energy generators and the primary sites of aerobic respiration, they provide most of the energy required for organ physiology. The kidney has the second-highest mitochondrial content and oxygen consumption in the human body, and mitochondrial function has been shown to play a crucial role in kidney development and disease progression 4. To maintain mitochondrial integrity and homeostasis, cells have evolved a variety of quality-control mechanisms, including mitochondrial DNA (mtDNA) repair, mitochondrial dynamics, mitophagy, and mitochondrial biogenesis 5. Among these, mitophagy, a highly conserved process in eukaryotic cells that selectively eliminates damaged or superfluous mitochondria via autophagy, has attracted increasing research attention due to its essential role in maintaining mitochondrial homeostasis 6. Consistent with the vital role of mitochondria in renal physiology, dysregulated mitophagy has been implicated in a growing number of kidney diseases, including acute kidney injury (AKI), chronic kidney disease (CKD), and diabetic kidney disease (DKD) 7-9. Despite this growing interest in investigating the mechanisms and potential targets of mitophagy in kidney diseases, the functions of many mitophagy-related targets remain incompletely understood.

In view of this, we provide a comprehensive review of recent findings on the role of mitophagy in kidney diseases and identify potential strategies to target mitophagy in their treatment. This review offers valuable insights into identifying potential targets in kidney disease, developing effective therapeutic strategies, and implementing them in clinical settings.

## 2. Mitophagy and mitochondrial homeostasis

Autophagy is an intracellular system responsible for waste disposal and recycling, playing a crucial role in maintaining cellular homeostasis. It facilitates the removal of pathological protein aggregates and helps cells cope with stresses 10. Through autophagy, a broad range of cytoplasmic components, including nucleic acids, proteins, and organelles, are degraded and transported to lysosomes, which function in the removal of specific organelles 11.

The autophagy process comprises four distinct stages: initiation, formation of the isolating membrane and autophagosome, fusion of the autophagosome with the lysosome, and the degradation of the autophagosome contents. Autophagy-related proteins (ATGs) are involved at various stages of this process 12. Mitophagy, as a selective form of autophagy, specifically targets damaged mitochondria exhibiting mtDNA injuries, lost membrane potential, and oxidative protein accumulation through various mitophagy pathways 13.

### 2.1. Classical mitophagy pathways

**Figure 1:**
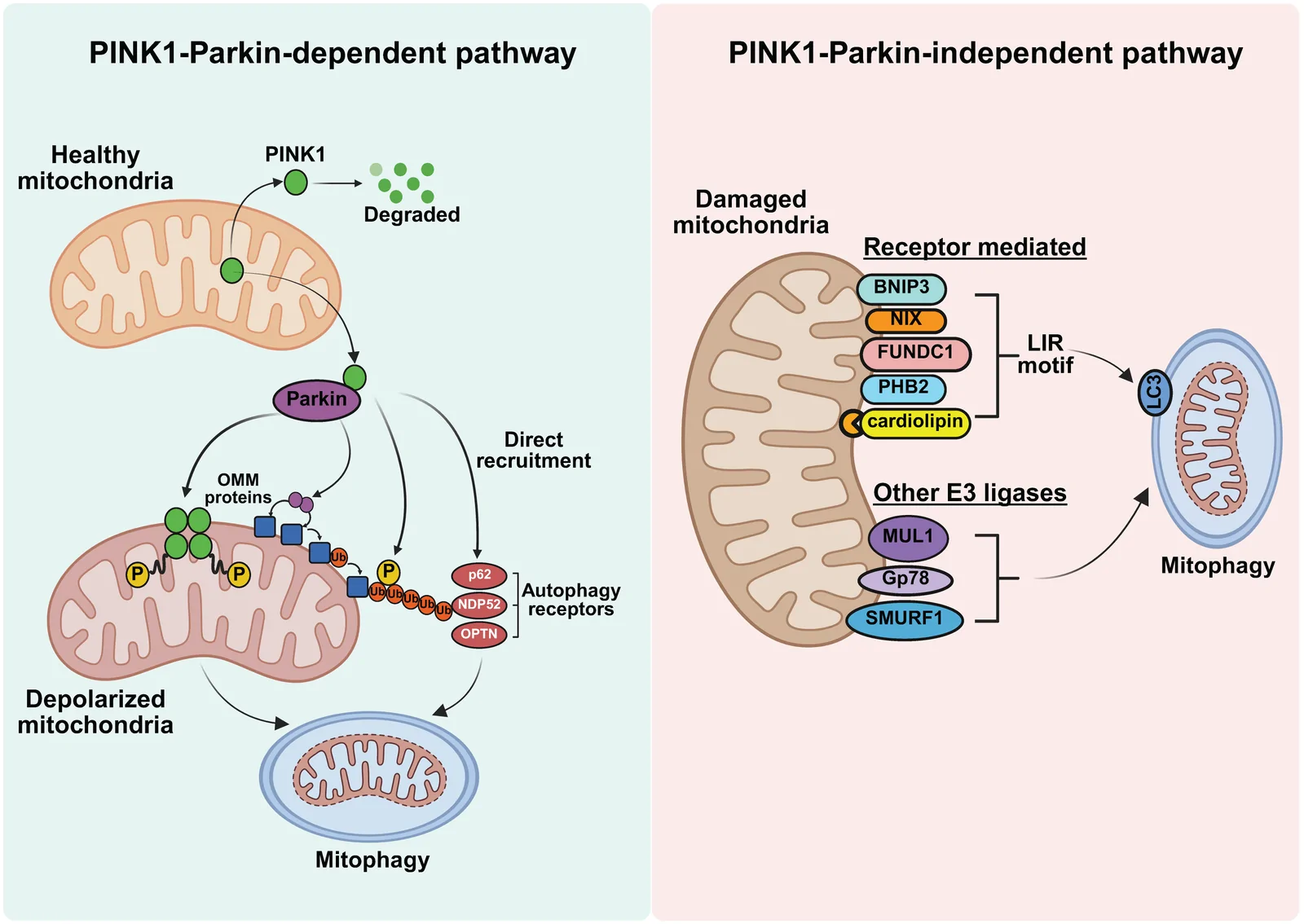
The mechanisms of mitophagy. Two main pathways: PINK1-Parkin-dependent pathway and PINK1-Parkin-independent pathway.

Mitophagy has attracted significant research interest since it was first proposed in 2005 14. The mechanisms of mitophagy can be broadly categorized into two pathways: the PINK1-Parkin-dependent pathway and the PINK1-Parkin-independent pathway 15 (**Figure 1**).

#### 2.1.1 PINK1-Parkin-dependent pathway

The dependent pathway relies on extensive ubiquitination of mitochondrial surface proteins to facilitate mitophagy. The primary proteins involved are PTEN-induced putative kinase 1 (PINK1) and Parkin, forming the PINK1-Parkin-mediated mitophagy pathway 16. PINK1 is a serine/threonine kinase located on depolarized mitochondria, and Parkin serves as an E3 ubiquitin ligase, facilitating the conjugation of ubiquitin to mitochondrial substrates 17. Under normal conditions, the PINK1 precursor protein in the cytoplasm is imported into healthy mitochondria by its N-terminal mitochondrial-targeting sequences (MTSs), where it is cleaved by mitochondrial proteases in the matrix and inner membrane. The cleaved PINK1 is then degraded in the cytoplasm through the ubiquitin-proteasome system. However, when mitochondrial membrane potential (MMP) diminishes, the entry of PINK1 precursor protein into mitochondria is blocked, leading to its accumulation on the outer mitochondrial membrane (OMM). This accumulation triggers PINK1 dimerization and autophosphorylation, activating its kinase activity. Activated PINK1 recruits Parkin from the cytoplasm to the OMM and initiates its E3 ubiquitin ligase activity. Parkin then ubiquitinates various OMM proteins, and these ubiquitin chains are subsequently phosphorylated. The phosphorylated ubiquitin-modified proteins on the OMM are recognized by autophagy adaptor proteins, such as p62, nuclear dot protein 52 (NDP52), and optineurin (OPTN), which act as "eat me" signals to initiate autophagy 18-20. Moreover, in addition to the PINK1-Parkin pathway, there are Parkin-independent ubiquitin-dependent pathways. It has been reported that PINK1 can directly recruit autophagy adaptor proteins to the mitochondria via ubiquitin phosphorylation, thereby promoting mitophagy 21. Notably, PINK1 can amplify mitophagy by generating phosphorylated ubiquitin, which enhances the recruitment and activation of downstream effectors 22.

#### 2.1.2 PINK1-Parkin-independent pathway

PINK1-Parkin-independent mitophagy is primarily mediated by mitophagy receptors that directly interact with autophagic machinery. Unlike the ubiquitin-dependent pathway, this process involves mitophagy receptors that contain a conserved microtubule-associated protein 1 light chain 3 (LC3)-interacting region (LIR). These receptors, including BCL2 interacting protein 3 (BNIP3), BCL2 interacting protein 3 like (BNIP3L, also known as NIX), FUN14 domain-containing 1 (FUNDC1), prohibitin 2 (PHB2), and cardiolipin, can directly bind to ATGs (e.g., LC3) via the LIR motif to initiate autophagosome formation 23, 24. In addition, other E3 ligases involved in mitophagy include Smad ubiquitination regulatory factor 1 (SMURF1), mitochondrial E3 ubiquitin protein ligase 1 (MUL1), and Gp78 25. The main mitophagy receptors and E3 ligases identified so far are listed in the table below (**Table 1**). These mitophagy regulators sense mitochondrial damage and facilitate mitophagy independently of PINK1-Parkin, often responding to specific signals or stress conditions.

### 2.2. Mitophagy in renal physiology

**Figure 2:**
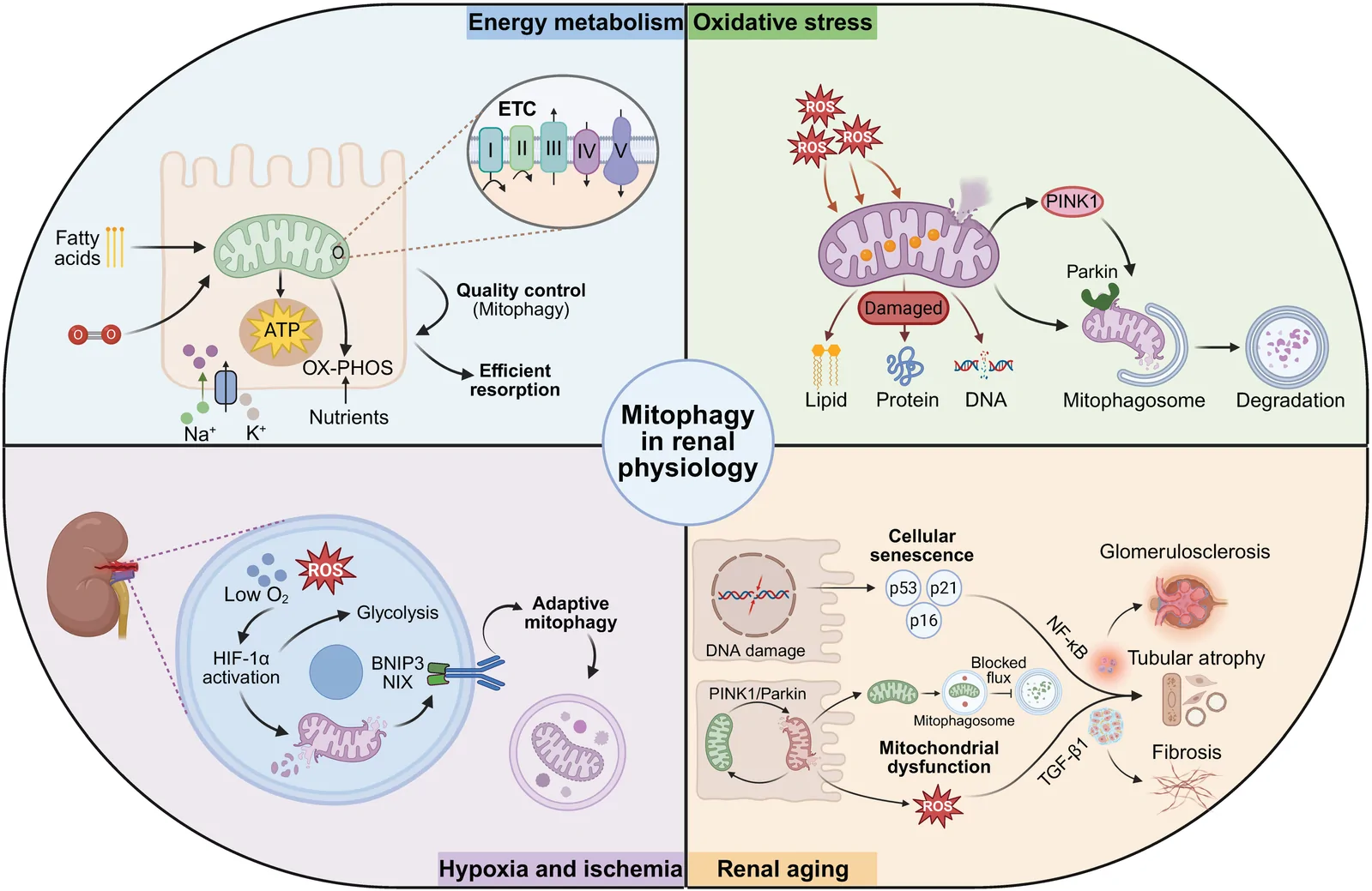
Mitophagy in renal physiology. Mitophagy is involved in various physiological processes of the kidney, such as energy metabolism, oxidative stress, hypoxia-ischemia, and aging.

The kidney is a vital organ within the human urinary system, responsible for filtering metabolic waste products and reabsorbing essential nutrients 26. This active excretion and reabsorption require substantial energy, primarily supplied by mitochondrial oxidative metabolism 27. Consequently, mitophagy, a key component of mitochondrial quality control (MQC), plays an important role in maintaining renal physiological functions (**Figure 2**).

#### 2.2.1 Energy metabolism and homeostasis

Mitochondria power renal proximal tubules, which require high energy for nutrients and ion reabsorption 28. These cells primarily rely on fatty acid oxidation (FAO) and oxidative phosphorylation (OX-PHOS) to produce ATP via the electron transport chain (ETC) 29. However, in renal medullary regions, particularly within distal tubules and collecting ducts where oxygen tension is relatively low, glycolysis serves as the predominant energy-producing pathway, ensuring cellular function under hypoxic conditions 30.

FAO and OX-PHOS are tightly linked mitochondrial energy metabolism processes. Long-chain fatty acids are transported into mitochondria through the carnitine transport system, where they undergo β-oxidation in the mitochondrial matrix to generate acetyl coenzyme A (acetyl-CoA), as well as the reduced electron carriers-nicotinamide adenine dinucleotide (NADH) and flavin adenine dinucleotide (FADH₂). Acetyl-CoA then enters the tricarboxylic acid cycle, leading to further production of NADH, FADH₂, and GTP/ATP. Subsequently, NADH donates electrons to complex I of the electron transport chain, whereas FADH₂-derived electrons enter through complex II or related flavoprotein-dependent pathways. Electron transfer through the respiratory chain, including complexes I-IV, drives proton pumping across the inner mitochondrial membrane (IMM), thereby generating an electrochemical proton gradient. Finally, ATP synthase, also known as complex V, uses this gradient to synthesize ATP 31.

Mitophagy plays a critical role in MQC during these metabolic processes. In response to mitochondrial damage, such as a decline of MMP, or an excessive production of reactive oxygen species (ROS), mitophagy-related signaling pathways including PINK1/Parkin, BNIP3, and FUNDC1 are activated to facilitate the selective removal of damaged mitochondria 32.

This targeted clearance prevents dysfunctional mitochondria from impairing the efficiency of FAO and OXPHOS, thereby preserving a healthy mitochondrial population, optimizing ATP synthesis, and maintaining the homeostasis of proximal tubule cells 33.

#### 2.2.2 Oxidative stress

Oxidative stress occurs when ROS production exceeds the capacity of antioxidant defenses. At physiological levels, ROS participate in the regulation of cellular signaling, metabolism, and immune responses, but excessive ROS can induce lipid peroxidation and damage cellular membranes, proteins, and DNA, thereby contributing to kidney injury 34. To maintain mitochondrial homeostasis, mitophagy is activated under oxidative stress to remove damaged mitochondria. Excessive ROS can also impair mitochondrial function and reduce MMP, triggering PINK1 accumulation on the outer mitochondrial membrane. Activated PINK1 subsequently phosphorylates ubiquitin and Parkin, promoting Parkin recruitment and activation, and promoting the ubiquitination of outer mitochondrial membrane proteins, thereby initiating the PINK1-Parkin-mediated mitophagy 35, 36. This process is essential for MQC and plays a protective role in kidney diseases, such as AKI and CKD.

#### 2.2.3 Response to hypoxia and ischemia

Hypoxia increases mitochondrial ROS (mtROS) production, in part due to electron leakage from respiratory chain complexes I and III and the accumulation of reducing equivalents such as NADH 37. Elevated ROS levels can disrupt redox hemostasis and contribute to the activation of hypoxia-responsive signaling pathways. Hypoxia-inducible factor (HIF) is primarily driven by oxygen limitation through stabilization of hypoxia-inducible factor 1, alpha subunit (HIF-1α), while mtROS may further modulate this response. Activated HIF promotes adaptive metabolic and quality-control programs including enhanced glycolysis and mitophagy 38. In renal cells, hypoxia or ischemia-reperfusion injury causes oxidative damage to proteins, lipids, and mitochondrial components, impairing ETC activity and membrane integrity 39. Under these conditions, mitophagy is primarily mediated by HIF-inducible receptors such as BNIP3 and NIX, which act as ubiquitin-independent mitophagy receptors by linking damaged mitochondria to LC3/GABARAP-positive autophagosomes for lysosomal degradation, thereby maintaining MQC and supporting cellular homeostasis 40, 41.

#### 2.2.4 Renal aging

The kidney is highly susceptible to aging, characterized by structural and functional changes such as glomerulosclerosis, tubular atrophy, and interstitial fibrosis 42. Cellular senescence drives these changes through DNA damage responses, p53/p21 and p16^INK4a/Rb^ signaling, NF-κB-mediated inflammation, and transforming growth factor β1 (TGF-β1)-induced fibrosis 43. Mitochondrial dysfunction is a hallmark of aging, involving impaired mitophagy, mtDNA damage, disrupted dynamics, and metabolic imbalance 44. According to the mitochondrial free radical theory of aging (MFRTA), the accumulation of oxidative stress impairs mitochondrial function and mitophagy, leading to decreased efficiency of OX-PHOS and elevated production of ROS 45. Although aging can activate mitophagy through pathways such as PINK1-Parkin, the concomitant blockade of autophagic flux prevents the clearance of damaged mitochondria. As a result, dysfunctional mitochondria accumulate, perpetuating oxidative stress and metabolic imbalance, thereby accelerating renal aging and functional decline 46.

## 3. Mitophagy and acute kidney injuries

**Figure 3:**
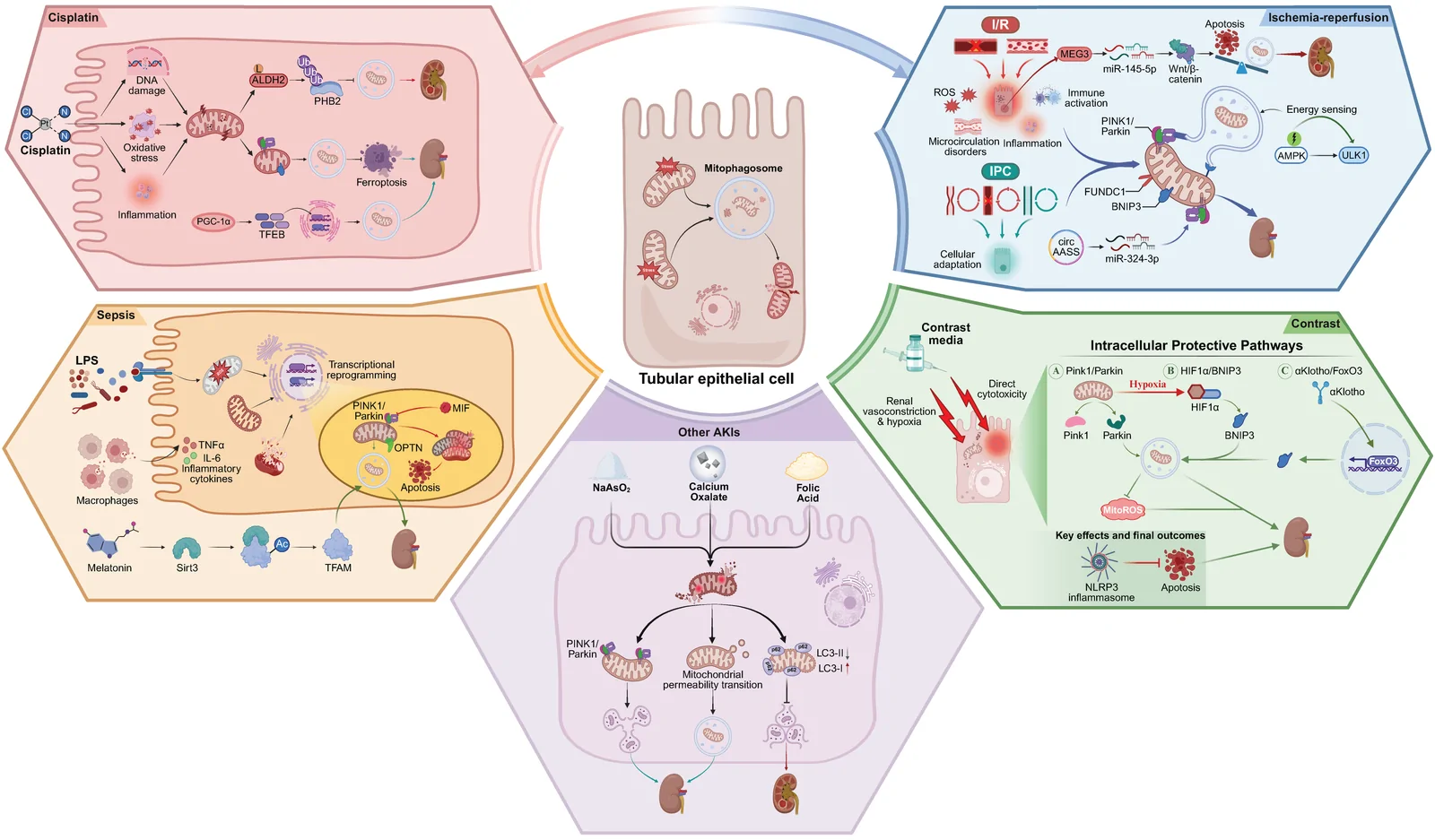
Mitophagy in AKI. AKI profoundly impairs mitochondrial homeostasis, leading to energy metabolism disorders, oxidative stress, and apoptosis. Concurrently, AKI induces activation of mitophagy to eliminate damaged mitochondria, and insufficient or excessive mitophagy further aggravates kidney injury.

Acute kidney injury is a clinical syndrome characterized by a rapid deterioration of renal function over a short period, caused by a variety of etiological factors. It manifests as a reduction in glomerular filtration rate, accumulation of creatinine and urea nitrogen, and disturbances in water, electrolyte, and acid-base homeostasis 47. Factors such as ischemia/reperfusion, exposure to surgical contrast agents, improper medication use, rhabdomyolysis, and infections can lead to AKI, many of which are known to stimulate mitophagy. Accumulating evidence suggests that mitophagy exerts a protective function, notably through the PINK1-Parkin and the BNIP3-mediated pathways 48, 49. Interestingly, some studies have also reported that mitophagy aggravates AKI 50. The discrepancy indicates the multiple roles of mitophagy in AKIs. Here, we review the role of mitophagy in different types of AKI and elucidate the mechanism underlying its activation in the kidney (**Figure 3**).

### 3.1. Ischemia-reperfusion-related AKI

Ischemia-reperfusion injury (IRI) denotes the exacerbation of tissue following the restoration of blood flow after a period of ischemia. In clinical settings, AKI caused by IRI is associated with a high mortality rate 51. The underlying mechanisms are intricate, including oxidative stress, endothelial and microcirculatory disorders, inflammation and immune activation, and various forms of controlled cell death (apoptosis, necroptosis, pyroptosis, and ferroptosis) 52. Studies show that ischemia/reperfusion (I/R) significantly induces oxidative damage in the kidney, resulting in renal tubular injury and alterations in mitochondrial ultrastructure 53. Growing research highlights the pivotal role of mitophagy in I/R-induced AKI. For example, renal IRI is exacerbated in mice with both single and double knockout of *Pink1* and *Parkin*, indicating that PINK1-Parkin-mediated mitophagy plays a protective role in ischemic AKI 54. Additionally, knockout of *Bnip3*, a specific mitophagy receptor, is shown to exacerbate injury during I/R-induced AKI *in vivo* 55. Other researchers found that overexpression of *BNIP3* restored hypoxia/reoxygenation (H/R)- induced reductions in mitophagy and mitigated cellular damage *in vitro*. These findings support the protective role of BNIP3-mediated mitophagy in renal IRI 56. Moreover, a recent study showed that *circAASS* alleviates I/R-induced AKI by improving mitochondrial homeostasis and inhibiting apoptosis and inflammatory response of renal tubular epithelial cells (RTECs). Specifically, the *circAASS* in the cytoplasm acts as a competitive endogenous RNA (ceRNA) by binding to *MIR324-3p*, thereby promoting PINK1 expression and enhancing mitophagy 57.

Ischemic preconditioning (IPC) involves a brief, intermittent period of I/R that induces adaptive responses in affected tissues, such as alterations in energy metabolism, reduced free radical production, and decreased inflammation and apoptosis 58. Studies have demonstrated that IPC confers protection against subsequent renal I/R in mice, primarily through PINK1-Parkin-dependent mitophagy 59. Furthermore, the study found that the renal protective effects of IPC against renal injury, inflammation, and tubular cell death are abolished in proximal tubule-specific *Fundc1* (a mitophagy receptor) knockout mice, highlighting the essential protective role of mitophagy in IPC 60. Additional studies have linked energy-sensing pathways to the regulation of mitophagy. In both *in vivo* and *in vitro* experiments, the phosphorylation level of Thr^172^- AMP-activated protein kinase (AMPK) α decreases rapidly after I/R. Mice with tubular epithelial cell-specific *AMPKα* deficiency exhibit more severe renal injury and increased apoptosis under I/R, partly because reduced AMPK activity inhibits UNC51-like kinase 1 (ULK1)-mediated autophagy, impairing the clearance of dysfunctional mitochondria 61. Contradictory findings also exist. c-MYC-activated maternally expressed 3 (MEG3) exacerbates renal IRI by upregulating rhotekin (RTKN) to induce mitophagy and trigger the Wnt/β-catenin pathway 62. The discrepancy may be explained by the fact that Wnt/β-catenin pathway activation can also promote apoptosis of tubular epithelial cells during AKI, with the pro-apoptotic effects potentially outweighing the protective benefits of mitophagy 63.

### 3.2. Contrast-induced AKI

Contrast media have been widely used to enhance the visibility of internal structures, organs, and tissues in medical imaging. Contrast-induced AKI (CI-AKI) occurs in more than 30% of patients following intravenous iodine administration, and currently, there are no effective targeted therapies 64. Contrast media have direct cytotoxicity leading to cellular death and tubular injury, while contrast media also alter renal hemodynamics, leading to renal vasoconstriction and renal hypoxia 65. Studies demonstrated that PINK1-Parkin-mediated mitophagy plays a crucial role in preventing apoptosis of RTECs by decreasing mtROS levels, which in turn reduces the activation of the NOD-like receptor thermal protein domain associated protein 3 (NLRP3) inflammasome in CI-AKI 66. The research group also revealed that upregulation of HIF-1α-BNIP3-mediated mitophagy attenuates NLRP3 inflammasome-mediated apoptosis in CI-AKI 67. Similar results were observed in that αKlotho promotes BNIP3-mediated mitophagy by activating forkhead box O3 (FOXO3), consequently reducing mtROS, cell apoptosis, and renal injury in CI-AKI 68. Collectively, these findings suggest that mitophagy forms a positive feedback loop in protecting against CI-AKI, highlighting its potential as a therapeutic target in drug development.

### 3.3. Mitophagy in sepsis-associated AKI

Sepsis-associated AKI (SA-AKI) is a sudden loss of kidney function due to the body's extreme response to infection, common in critically ill patients 69, 70. Sepsis-associated AKI progresses through four stages: intense inflammation (initiated by innate immune response), self-amplification (to exert antiviral response), maladaptive metabolic shutdown (transcriptional reprogramming), and recovery (metabolic adaptation) 71. Ultrastructural mitochondrial alterations, such as cristae disruption and mitochondrial swelling, are frequently observed during SA-AKI. In response, mitophagy is activated to minimize cellular damage and promote recovery 72. Mitophagy is activated, as demonstrated by the upregulation of PINK1-Parkin in mouse models of SA-AKI induced by lipopolysaccharide (LPS) or cecal ligation and puncture (CLP), as well as in human renal proximal tubular epithelial cells (HK-2) exposed to LPS. Nonetheless, knockout of *Pink1* or *Park2* impairs mitophagy by blocking mitochondrial accumulation of the autophagy receptor OPTN, resulting in exacerbated kidney injury and increased apoptosis 73. Alternatively, macrophage migration inhibitory factor (MIF) interferes with PINK1-Parkin interaction, affecting Parkin's mitochondrial recruitment and thereby impairing mitophagy 74. Researchers analyzed 48 patients with clinical sepsis and found that the extent of mitochondrial damage in renal tubular epithelial cells inversely correlates with recovery from AKI. They further found that melatonin, a potent SIRT3 (sirtuin 3) agonist, alleviates SA-AKI by promoting mitophagy via SIRT3-mediated deacetylation of transcription factor A, mitochondrial (TFAM), offering promising therapeutic insights 75.

### 3.4. Cisplatin-induced AKI

Cisplatin is an effective chemotherapeutic agent used to treat various solid tumors. However, its clinical application is limited by significant nephrotoxicity 76. Cisplatin has been shown to cause DNA/mitochondrial damage, oxidative stress, endoplasmic reticulum (ER) stress, inflammation (cytokines/immune cells), mitochondrial dysfunction (ATP loss, calcium imbalance), metabolic reprogramming (glycolysis, impaired FAO), and cell death (apoptosis, necroptosis, ferroptosis, etc) 77. Evidence indicates that mitophagy exerts protective effects in cisplatin-induced AKI 78. For instance, BNIP3 and PINK1-Parkin-mediated mitophagy can protect renal tubular epithelial cells from ferroptosis induced by cisplatin, primarily via the ROS/HO1/GPX4 signaling pathway 79. However, another study has shown that the deficiency of *Pink1* can improve acute kidney injury induced by cisplatin, possibly by inhibiting mitochondrial fission mediated by dynamin-related protein 1 (DRP1) and excessive mitochondrial phagocytosis 80. In addition, a recent study found markedly elevated levels of lactylation, a post-translational modification (PTM), in kidneys of AKI patients and in cisplatin-induced mice. Further studies showed that aldehyde dehydrogenase 2 (ALDH2) lactylation facilitates the ubiquitination and proteasome degradation of PHB2, a vital mitophagy receptor, thereby suppressing mitophagy and aggravating mitochondrial dysfunction 81. Conversely, a separate study reported that cisplatin inhibits mitophagy in renal tubule cells. However, peroxisome proliferator-activated receptor γ coactivator-1α (PGC-1α) reactivated mitophagy and lysosomal biogenesis via the transcription factor EB (TFEB), ultimately ameliorating kidney injury 82. These conflicting findings may suggest that mitophagy plays distinct, context-dependent roles at different stages of AKI.

### 3.5. Other AKIs

In clinical practice, several less common factors can induce AKI, including arsenic exposure, folic acid (FA) toxicity, and calcium oxalate crystal deposition. The role of mitophagy in AKI triggered by these factors is not fully understood. Exposure to NaAsO_2_ remarkably exacerbates renal tubular injury and mitochondrial dysfunction and increases the expression of mitophagy-related proteins. This study also demonstrated that PINK1-Parkin-dependent mitophagy exerts a protective effect against NaAsO2-induced AKI, but the precise underlying mechanism has yet to be elucidated 83. In a rat model of FA-induced AKI, the expression of PINK1 and p62 increases, while LC-3II/I decreases, suggesting that mitophagy is impaired 84. Additionally, AKI caused by calcium oxalate crystal deposition in distal renal tubular epithelial cells is associated with mitochondrial alterations characterized by mitochondrial permeability transition, which ultimately triggers mitophagy 85.

## 4. Mitophagy in chronic kidney disease

Chronic kidney disease (CKD) is characterized by persistent structural and functional renal abnormalities over three months 86. Common causes include diabetes mellitus, glomerulonephritis, and polycystic kidney disease, among others 87. Despite extensive research, the precise mechanisms underlying CKD development remain incompletely understood. Renal inflammation and fibrosis are common pathogenic mechanisms in CKD 88. Accumulating evidence indicates that mitochondrial dysfunction in renal tubular epithelial cells plays a crucial role in CKD progression and is closely linked to renal fibrosis 89. Studies have shown that the deficiency of acyl-CoA synthetase family 3 (*Acsm3*), involved in the metabolism of medium-chain fatty acids (MCFA), leads to metabolic disorders, which in turn disrupt mitochondrial homeostasis and ultimately aggravate unilateral ureteral obstruction (UUO)-induced renal fibrosis 90. Studies have demonstrated that mitophagy is induced in tubular epithelial cells during renal fibrosis. Under UUO or hypoxic conditions, deletion or silencing of *Bnip3* resulted in significantly increased mtROS production, mitochondrial damage, NLRP3 inflammasome activation, and elevated levels of α-smooth muscle actin (α-SMA) and TGF-β1, indicating that BNIP3-mediated mitophagy exhibits a protective effect against fibrosis 91. Furthermore, the knockout of *Parkin* inhibits mitophagy in endothelial cells, leading to an increase in interleukin-6 production, thereby inducing epithelial-to-mesenchymal transition (EndoMT), and ultimately exacerbating interstitial fibrosis in renal allografts of rats subjected to allogeneic kidney transplantation 92. Nevertheless, another study indirectly indicates that overactivation of mitophagy might cause a buildup of autophagosomes, hindering the efficient degradation of damaged mitochondria. This process instead results in the release of more mtDNA, triggers the stimulator of interferon genes (STING) pathway, and worsens inflammation and fibrosis 93. Of particular interest, an earlier study reported reduced levels of Parkin and autophagy in the renal tissues of UUO mice, in macrophages treated with TGF-β1, and in kidney tissues from adenine diet-induced CKD models 94, 95. The discrepancy suggests that alterations in mitophagy may differ across various cell types and disease stages. Nonetheless, all the evidence indicates that the dysfunction of mitophagy is an important factor contributing to the acceleration of renal fibrosis.

In summary, the prevailing view is that mitophagy plays a protective role in renal fibrosis. By reducing mitochondrial damage, limiting epithelial cell apoptosis, and decreasing inflammatory infiltration, mitophagy can mitigate tubulointerstitial fibrosis. However, it is important to recognize that models such as UUO, which induce rapid and irreversible fibrosis, may not fully capture the complexities of renal repair processes.

## 5. Mitophagy and diabetic kidney disease

Diabetic kidney disease (DKD) is characterized by persistent proteinuria and a gradual decline in glomerular filtration rate (GFR) attributable to prolonged diabetes, representing one of the most common microvascular complications of diabetes 96. Hyperglycemia is the primary driving pathogenesis in diabetic kidney disease, including hypertension, glomerular cell injury, disrupted tubular feedback mechanisms, renal hypoxia, inflammation, mitochondrial dysfunction, and impaired autophagy. These disturbances collectively promote podocyte loss, extracellular matrix expansion, glomerular sclerosis, and progressive renal function decline 97.

Bulk gene expression analyses have revealed several major dysregulated pathways in DKD, notably alterations in FAO and OXPHOS 98. Mitochondrial abnormalities are increasingly recognized as the central contributors to these changes. Studies have shown that mitophagy in the podocytes of patients with DKD is severely inhibited 99. Similarly, this phenomenon has also been observed in animal models of glomerular disease induced by high-fat diet (HFD) and streptozotocin (STZ) injection 100, 101. Besides, impaired mitophagy has been observed in the kidneys of diabetic patients and in RTECs derived from mice exposed to high glucose (HG), suggesting that mitochondrial dysfunction plays a pivotal role in the onset and progression of DKD 102.

One study revealed that the deficiency of *Ulk1* exacerbates kidney damage in diabetic mouse models. From a mechanistic perspective, the upregulation of X-linked inhibitor of apoptosis (XIAP) leads to the degradation of ULK1 through K48-linked polyubiquitination, thereby inhibiting mitophagy and disrupting carnitine metabolism. Restoring ULK1 protein expression by administering the ULK1 agonist echinacoside and supplementing with L-carnitine improves mitophagy and carnitine homeostasis, thereby alleviating DKD 103. Furthermore, tumor necrosis factor alpha-induced protein 8-like 1 (TIPE1) has been shown to mediate ubiquitination and subsequent proteasomal degradation of PHB2, disrupting mitophagy in RTECs under hyperglycemic conditions, consequently culminating in tubular injury and exacerbating DKD progression 104. Another study indicated that disrupted mitophagy and mitochondrial fission and fusion were associated with mtROS overproduction, MMP reduction, and renal interstitial fibrosis in diabetic rats. Notably, a Vitamin D receptor (VDR) agonist partially restored impaired mitophagy in diabetic rats via the mitofusin 2 (Mfn2)-mitochondria-associated ER membranes (MAMs) pathway, as evidenced by elevated levels of Pink1 and Parkin. From a mechanistic standpoint, VDR upregulates the expression of Mfn2, thereby reinstating the structural integrity of MAMs. This restoration of MAMs integrity facilitates the interaction between Fundc1 and LC3, which in turn triggers the activation of mitophagy 105. Studies have shown that inactivation of yes-associated protein 1 (YAP1) results in dysregulation of MQC and triggers abnormalities in mitochondrial biogenesis and mitophagy in renal tubular cells, ultimately leading to tubular damage in DKD 106. Additionally, Wang et al identified translocase of outer mitochondrial membrane 7 (TOMM7) as a critical regulator of mitophagy within renal tubules, contributing to the mitigation of DKD. Mechanistically, TOMM7 facilitates the recruitment of PLA2G6 to mitochondria. This process is accompanied by the restoration of mitochondrial membrane integrity, improved lipid homeostasis, and reduced ROS levels. Concurrently, TOMM7 promotes the stable accumulation of PINK1 on the OMM, thereby initiating the activation of mitophagy 107. Recent studies also have demonstrated that in HK-2 cells under HG/palmitic acid (PA) conditions, the nuclear receptor estrogen-related receptor alpha (ESRRA) transcriptionally activates autophagy-related protein 5 (ATG5) to support PINK1-dependent mitophagy and maintain tubular homeostasis 108.

**Figure 4:**
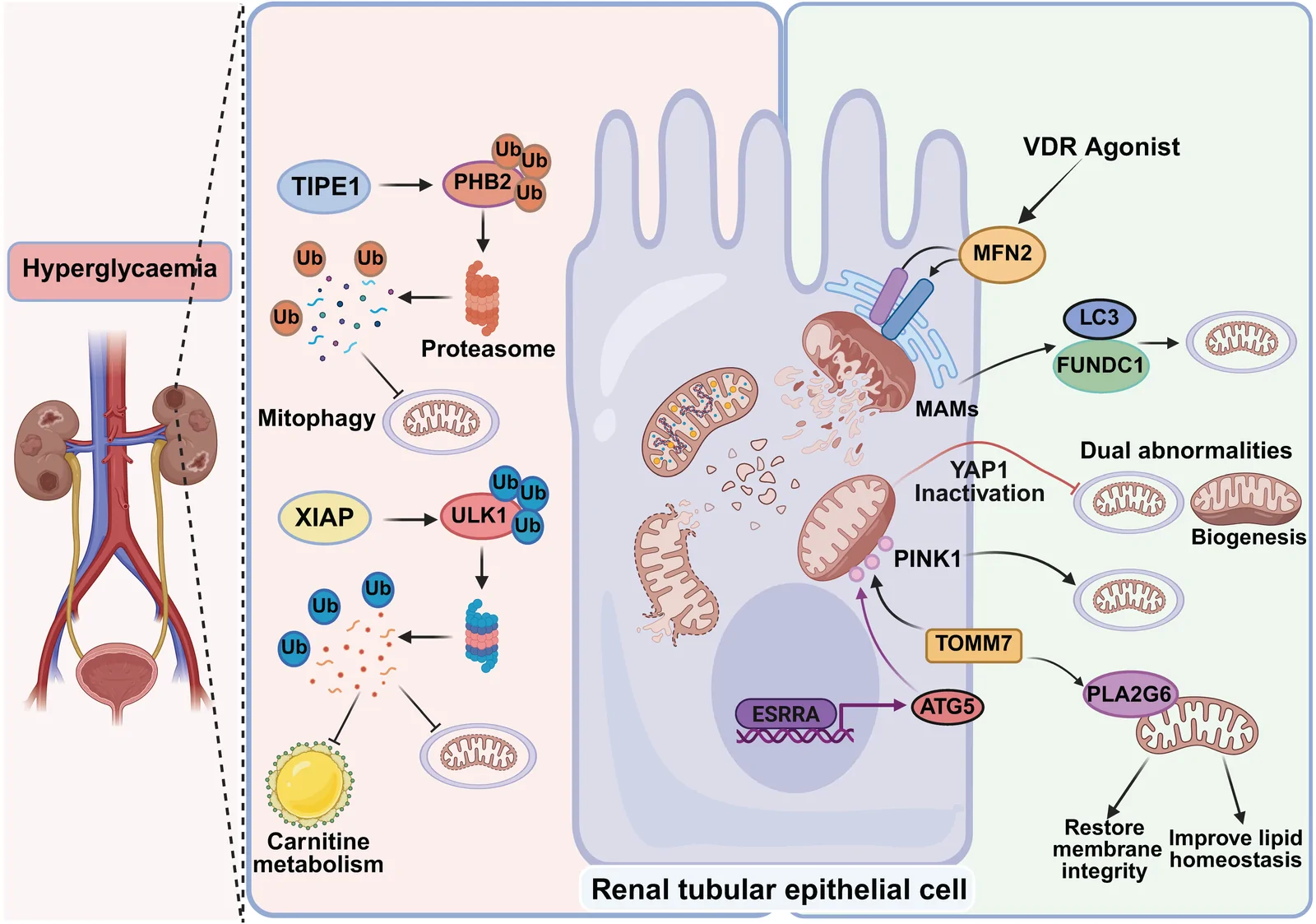
Mitophagy in DKD. In DKD, mitophagy is often inhibited, resulting in the accumulation of dysfunctional mitochondria. Conversely, proteins such as TOMM7, YAP1, and MFN2 promote mitophagy and improve mitochondrial function through distinct molecular pathways.

In summary, impairment of mitophagy and associated pathways, including ubiquitin-dependent and independent, is involved in ensuring MQC and sustaining the physiological function of mitophagy throughout DKD development (**Figure 4**). Nevertheless, the mechanisms by which impaired mitophagy contribute to glomerular and tubular injury in DKD remain incompletely understood, necessitating further mechanistic and translational research.

At this point, we have summarized the targets studied so far that mediate mitophagy in various kidney diseases, along with their potential functions and possible mechanisms (**Table 2**).

## 6. Targeting mitophagy for kidney diseases

### 6.1. Selective regulators of mitophagy

Numerous non-selective and selective mitophagy regulators have been identified, which modulate mitochondrial biological processes, such as FAO, OX-PHOS, ETC complexes, and mitophagy pathways 25. Metformin is a non-selective regulator that promotes mitophagy in peripheral blood mononuclear cells from patients with type 2 diabetes, primarily through the activation of AMPK 109. Several selective modulators have been developed to target the PINK1-Parkin signaling pathway. (**Table 3**). However, the druggability, clinical efficacy, and safety profiles of these modulators remain largely unknown.

### 6.2. Potential application for kidney diseases

Recently, mitophagy therapies targeting the kidneys have shown promise in cellular and animal models of kidney diseases.

#### 6.2.1 PINK1-Parkin-dependent pathway

The small-molecule compound UMI-77, identified by high-throughput screening, is a novel mitophagy inducer that enhances the interaction between myeloid cell leukemia 1 (MCL-1) and LC3A on the mitochondrial surface. Studies using UMI-77 to activate mitophagy in RTECs demonstrate that mitophagy activation mitigates mitochondrial dysfunction, inhibits the TGF-β/Smad signaling pathway, and alleviates inflammation, thereby delaying the development of renal fibrosis in UUO mice 110. Although UMI-77 has shown remarkable efficacy in preclinical animal studies, several potential obstacles remain before its clinical application can be realized. For example, MCL-1, the main target of UMI-77, not only regulates mitophagy but also plays critical roles in apoptosis, cell cycle progression, immune cell survival, and tumor biology. Therefore, pharmacological modulation of MCL-1 may produce unintended effects such as altered cell survival, immune dysregulation, or even oncogenic risk, which must be carefully evaluated in future studies 111. Additionally, as a BH3 mimetic, UMI-77 may induce cell apoptosis at high doses, thereby increasing the risk of tissue injury 112. Currently, systematic pharmacokinetic and toxicological data are still lacking, and further studies are needed to comprehensively evaluate its safety and translational potential. Furthermore, studies have found that the AMPK agonist metformin improves renal oxidative stress and tubulointerstitial fibrosis in diabetic mice by activating the p-AMPK-PINK1-Parkin mitophagy pathway 113. Lycopene inhibits the activation of the protein kinase B (also known as AKT) signal, activates mitophagy, and alleviates renal fibrosis 114. WJ-39, the novel aldo-keto reductase inhibitor, has been discovered to preserve the structural and functional integrity of renal tubules in diabetic nephropathy by activating PINK1-Parkin-mediated mitophagy 115. Furthermore, Urolithin A (Uro A) suppresses the inflammatory response mediated by STING-NLRP3 through inducing PINK1-Parkin-dependent mitophagy, thereby alleviating fructose-induced hypouricemic nephropathy 116. Uro A has advanced to the Phase I clinical trial, where oral administration in healthy adults improves immune function by modulating immune cell mitochondrial activity 117. However, the efficacy of Uro A in treating kidney diseases remains to be verified through large-scale clinical studies. Its clinical translation also faces several challenges, including limited tissue specificity, significant interindividual variability in bioavailability, and a lack of long-term safety data. These limitations may hinder its further clinical application. Ruxolitinib has been demonstrated to ameliorate renal fibrosis by activating PINK1-Parkin-mediated mitophagy 118. Recently, several other compounds, including MTK458, LCL768, and MTX115325, have been shown to modulate mitophagy, though their effects and mechanisms in kidney disease remain to be fully elucidated 119-121. The potential therapeutic application of these compounds in kidney diseases warrants further investigation.

Chinese medicine and phytochemicals also have great potential to modulate mitophagy in kidney disease. For example, *Huangkui* capsule induces mitophagy in renal tubular cells through upregulating PINK1 expression, thereby alleviating diabetic nephropathy 122. Similarly, swietenine has been reported to improve renal function in diabetic mice by enhancing mitophagy via the activation of the Acsf2/PHB2/PINK1 signaling pathway 123. In addition, naringenin promotes mitophagy by directly binding to PINK1 and enhancing its stability, thereby alleviating LPS-induced renal structural damage and oxidative damage 124.

#### 6.2.2 Receptor-mediated pathway

Resveratrol alleviates sepsis-induced acute kidney injury by activating SIRT1, promoting the upregulation of PGC-1α, and enhancing BNIP3/NIX-mediated mitophagy 125. Cobalt oxide-polyethylene glycol-triphenylphosphine (COPT) nanoparticles have been reported to ameliorate H/R-induced mitochondrial damage by enhancing BNIP3-mediated mitophagy both *in vitro* and *in vivo*. These nanoparticles also alleviate ischemic AKI in a mouse model and gentamicin-induced AKI in a zebrafish model 126. Additionally, a recent study suggests that melatonin can protect kidney function by regulating FUNDC1-mediated mitophagy 127.

### 6.3. Challenges in clinical application

Despite its therapeutic potential, the clinical application of mitophagy-targeted strategies in kidney disease still faces several challenges. First, mitophagy exhibits substantial cell-type and disease-subtype specificity, which complicates the development of personalized therapeutic approaches. For instance, activation of mitophagy in proximal tubular cells may exert protective effects, whereas excessive or maladaptive mitophagy in distal tubules or podocytes may contribute to cellular dysfunction, making it difficult to design uniform interventions. Second, variations in the agents, dosages, and delivery systems used to modulate mitophagy across different studies have resulted in inconsistent outcomes. This heterogeneity limits reproducibility and hinders the establishment of standardized treatment guidelines. More importantly, the long-term safety of mitophagy-targeted strategies remains to be fully determined, particularly in patients with severe kidney disease or immunosuppression. In addition, systemic delivery of mitophagy modulators raises concerns about off-target effects in non-renal tissues, such as the heart and liver 128. Finally, the potential for drug resistance or maladaptive cellular adaptation should not be overlooked. If cells adapt to chronic mitophagy stimulation, therapeutic effect may diminish over time, while persistent mitochondrial stress could create latent health risks.

## 7. Discussion and Conclusion

The role of mitophagy in kidney diseases is complex and context-dependent, exhibiting a bidirectional effect influenced by the specific pathological environment. In AKI, mitophagy protects renal tubular cells by eliminating damaged mitochondria and reducing ROS accumulation 50. In renal fibrosis, mitophagy suppresses EndoMT and thereby delays fibrotic progression; however, when excessively activated, mitophagy may promote mtDNA release and inflammatory responses, and the excessive clearance of mitochondria can further cause energy deficiency and structural abnormalities in renal tubular epithelial cells, ultimately leading to tubular atrophy and fibrosis 93, 114, 129. In DKD, podocytes and renal tubular cells highly rely on mitochondrial homeostasis, and enhancement of mitophagy, such as through AMPK activation, appears to exert protective effects 113. These findings highlight that future treatment strategies need to precisely regulate mitophagy levels based on the underlying disease mechanisms, rather than broadly enhancing or suppressing the process.

Furthermore, accumulating evidence indicates that the role of mitophagy varies across different kidney intrinsic cell types, including podocytes and endothelial cells 130. For instance, podocytes are particularly reliant on mitochondrial homeostasis, and deficiency in PINK1-Parkin-mediated mitophagy has been shown to exacerbate proteinuria and podocyte injury 131. By contrast, in human umbilical vein endothelial cells (HUVECs) induced by tumor necrosis factor-alpha (TNFα), suppression of the Rictor/mTORC2 signaling pathway enhances BNIP3-dependent mitophagy, thereby alleviating EndoMT and interstitial fibrosis in renal transplantation 132. Beyond intrinsic renal cells, immune populations such as macrophages and T lymphocytes also play pivotal roles in kidney pathogenesis, with emerging evidence suggesting that mitophagy regulates macrophage polarization and T cell activation 133-135. Collectively, these findings raise a compelling research question: Does mitophagy operate through distinct molecular pathways in podocytes, renal vascular endothelial cells, and immune cells, and do these cell-specific regulatory mechanisms ultimately shape the pathological classifications and therapeutic outcomes of kidney diseases? Addressing this question will require future investigations that focus on cell-type-specific mechanisms, integrating genetic models, single-cell approaches, and targeted delivery strategies to delineate the precise role of mitophagy in renal pathophysiology.

Emerging evidence indicates that mitophagy interacts intricately with multiple forms of cell death, thereby shaping the outcome of kidney injury 136. On the one hand, mitophagy mitigates apoptosis by removing damaged mitochondria, reducing ROS accumulation and preventing cytochrome c release. Conversely, insufficient mitophagy accelerates apoptotic signaling 50. In the context of necrosis, excessive or dysregulated mitophagy may precipitate energy collapse and membrane rupture, while necrotic inflammation can further impair autophagic flux 137. Mitophagy also intersects with pyroptosis, where suppression of mtROS and mtDNA release attenuates NLRP3 inflammasome activation, reducing inflammatory cell death. Moreover, links between mitophagy and ferroptosis have been highlighted, as mitochondrial iron handling and lipid peroxidation can couple mitophagy imbalance to ferroptotic injury 138. Taken together, these interactions underscore mitophagy as a central regulator of cellular fate in kidney diseases, functioning as either a protective mechanism or a potential pathological driver depending on the context.

Given that mitophagy is a ubiquitous cellular process, systemic administration of modulators may lead to widespread, non-targeted effects in other organs. Therefore, the targeted delivery strategy for the kidneys is critical for advancing therapeutic applications. Recent studies have demonstrated that nanoparticles encapsulating *Parkin* mRNA can promote synchronous regulation of functional and dysfunctional mitochondria, showing promise for treating pulmonary fibrosis 139. Simultaneously, researchers have recently developed a biomimetic high-density lipoprotein (bHDL) lipid nanoparticle that exhibits excellent targeting ability for damaged renal tubular epithelial cells through endocytosis mediated by kidney injury molecule-1 (KIM-1) 140. Based on these findings, we propose a testable hypothesis: The bHDL lipid nanoparticles that encapsulate *Parkin* mRNA could specifically target renal tissues and induce mitophagy, thus providing a potential strategy for the treatment of selected kidney diseases. Moreover, mitochondrial transplantation, an emerging cell therapy strategy, has been applied to treat experimental cerebellar degenerative diseases, in which mitochondria transplanted from the liver into the cerebellum of *Drp1* conditional knockout mice improve mitochondrial function, delay neuronal apoptosis, and alleviate cerebellar ataxia 141. These strategies offer valuable insights for developing targeted mitochondrial therapies in kidney diseases.

Additionally, some clinically used drugs indirectly modulate mitophagy. For instance, Metformin and other AMPK agonists help maintain mitochondrial homeostasis and have demonstrated protective effects in animal models of kidney diseases 109, 113. Future research should explore the potential of these drugs to improve mitophagy-related kidney disease, with a systematic evaluation of their efficacy, safety, and dosimetric characteristics to facilitate clinical translation.

In conclusion, mitophagy plays a multifaceted role in kidney diseases, exerting both protective and detrimental effects depending on disease type, cellular context, and immune involvement. Moving forward, therapeutic strategies should focus on precise regulation and targeted delivery, combined with mechanism research and translational exploration, ultimately opening new avenues for the treatment of various kidney diseases.

## Figures and Tables

**Table 1:** An overview of main PINK1-Parkin-independent mitophagy regulators

Protein or lipid	Property	Localization	Inducers	Regulators	Autophagic interactors	References
BNIP3	Receptor	OMM	Hypoxia	FOXO3↑ HIF1α↑ MA-5↑	LC3B Atg8	56, 68 142-144
BNIP3L/NIX	Receptor	OMM	Hypoxia High OXPHOS activity	HIF1α↑ FBXL4↓	GABARAPL1	145-148
FUNDC1	Receptor	OMM	Hypoxia FCCP	ULK1↑ PGAM5↑ CK2↓ SRC↓	LC3B	149-153
FKBP8	Receptor	OMM	Starvation	Rheb↓	LC3A	154-156
BCL2L13	Receptor	OMM	CCCP	-	LC3B	157
AMBRA1	Receptor	OMM	FCCP	MCL-1↓ IKKα↑ SRC↓ HUWE1↑	LC3 Beclin1	92, 158-162
NLRX1	Receptor	mtMatrix	CCCP	TRMT10C↓	LC3	163-166
PHB2	Receptor	IMM	CCCP oligomycin + antimycin	TIPE1↓ miR-24-3p↓ ALDH2↓	LC3B	81, 104, 167, 168
Cardiolipin	Receptor	IMM OMM	FCCP Rotenone Staurosporine 6-hydroxydopamine	CRLS1↑ SNCA↑ PLSCR3↑	LC3 Beclin1	169-171
NIPSNAP1/2	Receptor	mtMatrix	Hypoxia CCCP oligomycin + antimycin	ATMLP↓	LC3B Atg8	172, 173
SMURF1	E3-ubiquitin ligase	OMM	Hypoxia	TRIB3↓	LC3	174, 175
MUL1	E3-ubiquitin ligase	OMM	Selenite	miR-135b-5p↑	GABARAPL1	176, 177
Gp78	E3-ubiquitin ligase	ER membrane	CCCP	MGRN1↓	LC3	178, 179
SIAH1	E3-ubiquitin ligase	Cytoplasm Nuclear	-	PINK1↑	LC3	180
ARIH1	E3-ubiquitin ligase	Cytoplasm Nuclear	CCCP	PINK1↑	-	181

**Abbreviations:** AMBRA1: autophagy and beclin 1 regulator 1; ATMLP: lncRNA AFAP1-AS1 translated mitochondrial-localized peptide; ARIH1: ariadne RBR E3 ubiquitin protein ligase 1; AURKA: aurora kinase A; BCL2L13: BCL2 like 13; CCCP: carbonyl cyanide 3-chlorophenylhydrazone; CK2: casein kinase 2; CRLS1: cardiolipin synthase 1; FBXL4: F-box and leucine-rich repeat protein 4; FCCP: carbonyl cyanide 4-(trifluoromethoxy)phenylhydrazone; GABARAPL1: GABA type A receptor associated protein like 1; HUWE1: HECT, UBA and WWE domain containing E3 ubiquitin protein ligase 1; IKKα: inhibitory kappa B kinase α; LC3B: microtubule associated protein 1 light chain 3 beta; MA-5: mitochonic acid 5; MGRN1: mahogunin, ring finger 1; NIPSNAP1/2: nitrophenylphosphatase domain and non-neuronal SNAP25-like protein homolog 1/2; NLRX1: NLR family member X1; PLSCR3: phospholipid scramblase 3; Rheb: ras homolog, mTORC1 binding; SIAH1: siah E3 ubiquitin protein ligase 1; SNCA: alpha-synuclein; SRC: SRC proto-oncogene, non-receptor tyrosine kinase; TRIB3: tribbles pseudokinase 3; TRMT10C: tRNA methyltransferase 10C.

**Table 2:** Regulatory targets and potential functions of mitophagy in different types of kidney diseases

Kidney disease	Cause of disease	Regulatory target	Cell lines	Potential function	Possible mechanism	Reference
AKI	I/R	PINK1-Parkin	HK-2	Anti-AKI	ROS production↓ Inflammation↓	54
		BNIP3	BUMPT	Anti-AKI	Inflammatory cell infiltration↓ Release of proinflammatory cytokines↓	55
		BNIP3	HK-2	Anti-AKI	Cell apoptosis↓ ROS production↓	56
		Atg7	RPTCs	Anti-AKI	Susceptibility to damage↓ Tubular cell necrosis↓	59
		FUNDC1	PRTCs	Anti-AKI	Mitochondrial quality↑ Drp1-dependent mitochondrial fission↑	60
		ULK1	PRTCs	Anti-AKI	Fatty acid oxidation↓ Tubular cell apoptosis↓	61
AKI	Contrast	PINK1-Parkin	HK-2	Anti-AKI	mtROS production↓ NLRP3 inflammasome activation↓ Cell apoptosis and tissue damage↓	66
		BNIP3	HK-2	Anti-AKI	mtROS production↓ Apoptosis↓	67, 68
	Spesis	PINK1-Parkin	RPTCs	Anti-AKI	Inflammation↓ Apoptosis↓	73
		TFAM	HK-2	Anti-AKI	Mitochondrial respiratory chain↑ NAD/NADH balance↑	75
	Cisplatin	PINK1-Parkin	BUMPT	Anti-AKI	Renal functional loss↓ Tissue damage↓ Apoptosis↓	78
		PHB2	HK-2	Anti-AKI	Mitochondrial morphology/function↑ Mitochondrial apoptosis↓	81
		TFEB	mIMCD-3	Anti-AKI	mtROS production↓	82
AKI	Arsenic	PINK1-Parkin	HK-2	Anti-AKI	Mitochondrial biogenesis and fission↑ Inflammation↓	83
	Folic acid	PINK1-Parkin	LLC-PK1	Anti-AKI	Oxidative stress↓ Tubular injury and death↓ Inflammation and fibrosis↓	84
	Calcium oxalate	PINK1-Parkin	HK-2	Anti-AKI	Necroptosis↓ Interstitial inflammation↓	85
CKD	UUO H/R	BNIP3	HK-2	Anti-fibrotic	mtROS production↓ NLRP3 inflammasome↓	91
	UUO AD TGF-β1	PINK1-Parkin	BMDMs THP-1	Anti-fibrotic	mtROS production↓ Macrophage-polarization toward M2↑	94
DKD	HG	OPTN	mPTECs	Anti-DKD	mtROS production↓ Cellular senescence↓	102
	Single-side Nx+STZ	PHB2	HK-2	Anti-DKD	Mitochondrial morphology and MMP↑ EMT↓ Tubular cell death↓	104
DKD	*db/db* HFD+STZ HG/ TGF-β1	ULK1	mPTECs HK-2	Anti-DKD	mtROS production↓ MMP↑ Carnitine homeostasis↑	103
	*db/db* HG/PA	PINK1-Parkin	HK-2	Anti-DKD	mtROS production↓ MMP↑ Inflammation↓	182

**Abbreviations:** AD: adenine diet; Atg7: autophagy-related protein 7; BMDMs: bone marrow-derived macrophages; BUMPT: Boston University mouse proximal tubule; EMT: epithelial-mesenchymal transition; LLC-PK1: proximal tubular cell line derived from porcine kidney; mIMCD-3: mouse inner medullary collecting duct cell line; Nx: nephrectomy; PRTC: primary renal tubular epithelial cells; RPTC: renal proximal tubular cells; THP-1: human acute monocytic leukemia cells.

**Table 3:** Mitophagy-specific activators and modulators

Effect	Drug name	Drug type	Target	Mechanisms	Research progress	Reference
Activators	FB231	Small molecule	Parkin	Directly enhancing PINK1-Parkin-dependent mitophagy	Cell + animal	119
	MTK458	Small molecule	PINK1	Lowering the threshold for cells to respond to mitochondrial damage	Cell + animal	119
	T0467	Small molecule	Parkin	Promoting the translocation of Parkin to the damaged mitochondria	Cell-based	183
	PARL-IN-1/2	Small molecule	Parkin	Promoting the translocation of Parkin to the damaged mitochondria	Cell-based	183
	KTP	Small molecule	PINK1	Enhancing the kinase activity of PINK1	Cell-based	184
Modulators	BIO-2007817	Molecular glue	PINK1	Promoting the accumulation of PINK1 outside the damaged OMM and enhancing the phosphorylation of PINK1 by ubiquitin	Cell-based	185
	LCL768	Molecular glue	Parkin	Attenuating Parkin succination to promote Parkin activation	Cell-based	120

## Abbreviations

AKI: acute kidney injury
CKD: chronic kidney disease
DKD: diabetic kidney disease
MQC: mitochondrial quality control
ATGs: autophagy-related proteins
PINK1: PTEN-induced putative kinase 1
MMP: mitochondrial membrane potential
FAO: fatty acid oxidation
OX-PHOS: oxidative phosphorylation
ROS: reactive oxygen species
I/R: ischemia/reperfusion
OMM: outer mitochondrial membrane
NDP52: nuclear dot protein 52
OPTN: optineurin
ETC: electron transport chain
MFRTA: mitochondrial free radical theory of aging
RTECs: renal tubular epithelial cells
ULK1: UNC51-like kinase 1
